# The New York Surgical Society

**Published:** 1880-09

**Authors:** 


### Reports of Societies.

### THE NEW YORK SURGICAL SOCIETY.

*Ganglion.*—Dr. E. L. Keyes presented a patient, accompanied by a history as follows:

John L. F., physician, aged fifty, had good health until twenty-three years old, when he had typhoid fever, and two years afterward an attack of typhus.

At the age of twenty-four ganglia formed on the anterier aspect of both wrists, commencing about the same time, and followed shortly after by inflammation of the knuckle-joint of the left index finger. This finger-joint continued swollen so long that it was thought it must contain pus; an incision was made, followed by the discharge of a clear fluid. A tedious suppuration followed the incision, and a final cure by anchylosis, with nearly half an inch shortening af the finger.

The ganglions contained a fluid which could be pressed back and forth, from the wrist to the palm, attended with a crepitating feeling. During the fourth year of the right ganglion the fluid found its way into the sheaths of the flexor tendons of the thumb, little and right fingers; and during the fifth year of the left ganglion precisely the same thing took place in the left hand, leaving only the index and middle fingers of each hand free.

These ganglions were never attended with pain of any consequence—an uneasy sense of distention was all that was complained of; but the power of the hands was greatly diminished.

Various treatment was tried without obtaining relief. Temporary benefit was derived from rest, alternate hot and cold showering, and bandaging.

At the age of thirty-one, and seventh year of the right ganglion, the right wrist-joint was attacked with inflammation, long continued, and entirely disabling the hand for over two years. The recovery was by partial anchylosis and an apparent cure of the ganglion. Fourteen years later, at the age of forty five, and twenty-first year of the left ganglion, precisely the same thing took place on the left side—that is, long-continued inflammation of the left wrist-joint, followed by partial anchylosis and the apparent cure of the ganglion.

There is very little power in either hand.

In February, 1863, he was attacked with irritability of the bladder, probably caused by exposure during cold and wet weather. A partial recovery took place in about two weeks, but the bladder never fully recovered its former tolerance. Afterward there were occasional attacks of irritability, sometimes lasting a few days, and again only a few hours. These attacks were not troublesome at night during sound sleep, but if the night was a restless one the irritability was apt to be as great during the night as during the day. This condition lasted about ten years, or until the winter of 1872–73, when the irratability became permanent, and gradually increased in severity until January, 1875, when it apparently reached its height. Since that time the calls to micturate occur about every half-hour day and night.

Pain just before, sometimes during, and occasionally immediately after urination; but usually there is a feeling of relief after the act. Blood is frequently seen, generally intimately mixed, but sometimes, when there is tenesmus, only at the finish. Movements increase the irritability. During the last six years the urine has contained pus, sometimes blood, and is always acid.

The amount of urine passed each time is from one to two ounces, and there is about an equal amount of residual urine.

There is no urethral obstruction, no prostatic enlargement, no stone.

No joints other than those above mentioned have ever been affected.

He has had three attacks of lumbago confining him on back six or eight weeks each time.

The family history is unimportant.

Dr. Willard Parker remarked that he had opened ganglions repeatedly, and was not sure but that it was the only way in which to get rid of them, especially when they contained the little granules, rice-like bodies. He had seen the same condition about the ankle-joint.

The President remarked that he had had no experience with reference to spontaneous cure of ganglion. He then referred to a case in which there was a ganglion on the dorsal and palmar surface of the right hand, involving the tendons of the thumb and little finger. The patient accidentally stuck a pin into one of the bursæ running along the little finger, and the opening gave exit to a certain quantity of clear fluid. No inflammation followed and recovery took place. The patient, however, did not get well of his trouble, and was unable to flex his wrist without pain.

Dr. Sands had also seen several cases in which suppuration had followed an opening and the patients recovered.

Dr. F. D. Lente referred to a case of cure without operation. The patient was a delicate female of scrofulous habit, and the ganglia were due, apparently, to over exercise of the tendons of the hand. There were several swellings upon the back and the front of the hand, and there was one which gave a feeling of crepitation, and fluid could be squeezed from one compartment to another under the annular ligament. Dr. Lente operated upon one of the swellings, and it was relieved, but not entirely cured. The ganglion, however, which incapacitated her most, was seen by Dr. Parker, who advised against an operation. The patient was then placed on the use of syrup of the iodide of iron in teaspoonful doses three times daily, rest insisted upon, and soon afterward she passed from under observation for ten months, when she returned cured. She had taken no medicine save the iron, which had been continued regularly, and no other treatment was employed.

Dr. Parker referred to the first case that came under his observation, in the person of a lady twenty-two years of age. In that case he made an oblique opening, forced everything out, kept the hand still, made pressure, and complete recovery took place. The important question was, what causes these collections? It would seem that in Dr. Lente's case the swelling was the result of an impoverished condition of the system, together with a certain amount of inflammatory action about the bursæ, tendons, etc., and that when a certain stage was reached these concretions were formed.

Dr. E. Mason referred to two cases in which he had punctured the ganglions, and then used pressure with a compress and bandage. In one case the sac did not refill. The result in the other case he did not know.

Dr. Briddon referred to two cases in which he found the rice-like concretions. In one case the patient sometimes indulged in large quantities of stimulants, and when he did so the ganglion was more troublesome than at other times. It was left without special treatment. In the other case suppuration took place, which became profuse and was prolonged, and the patient died in consequence of amyloid degeneration of the internal organs.

The President then read a paper on "Perityphlitis."

It was based on twenty-six cases, which the author separated into four divisions, as follows:

1. *Cases terminating in resolution without evidence of suppuration.*—In this group there were ten cases, in all of which the disease disappeared without showing any signs of suppuration. In all of these cases the diagnosis was carefully and fairly made out.

2. *Cases of abscess terminating in spontaneous recovery.*—Of those there were three, and they proved that even when a perityphilitic abscess is left to itself, its course is not always unfavorable, but that its contents may be discharged into the intestine or possibly into the bladder, without any serious consequences.

3. *Cases of abscess treated by operation.*—In this group there were eleven cases, the study of which revealed many interesting facts. In every case that had fallen under Dr.

Sand's observation, the operation had been performed essentially in the manner recommended by Dr. Willard Parker, in the year 1867: "An incision several inches in length, and usually parallel with Poupart's ligament, was made over the most prominent part of the tumor, through the skin and subcutaneous fat. Subsequently the deeper layers were divided until the abscess was reached, or until the fascia transversalis came into view. If fluctuation then became evident, the abscess was immediately opened, otherwise the fascia was penetrated in various directions by means of a hypodermic syringe until the seat of the abscess is discovered, when the operation was completed by entering a narrow bistoury alongside of the needle." An external incision of two inches, Dr. Sands thought, would afford ample space.

4. *Cases of abscess unopened, and ending fatally.*—This group comprised only two cases, both of which possessed features of interest.

Of the twenty-six cases, three terminated fatally; one belonging to the third group, and two comprising the fourth group, in all other cases the patient recovering.

Dr. E. L. Keyes referred to three cases, two of which recovered without surgical interference. The other case presented certain points of interest, the most interesting of which were the following: A boy, seventeen years old, strained himself by lifting upon a rope; he at once felt that he was hurt in the right groin. Localized evidence of peritiphylitis developed, which disappeared gradually under the use of the usual remedies, but recurred at the end of six months, again subsided, recurred the second time and again subsided, but appeared again, and was then opened by Dr. Van Buren after extensive suppuration had occurred. After it was opened more pus formed and burrowed to a considerable extent. There was escape of fetid gas from the opening, but no fæcal matter. The boy was sick for a long time, but finally rallied and became quite well. At the end of a year, without known cause, another attack was developed, preceded by an intense chill. Suppuration occurred quickly; fluctuations at the situation of the old cicatrix was detected. The swelling was opened and exit given to fetid material and gas, but no fæcal matter, and recovery took place within a reasonably short period. Another interval occurred, and again, after being perfectly well apparently, the patient was seized with a severe chill that lasted about half an hour; tenderness developed at the site of the old cicatrix, and on the following morning fluctuation was detected. The swelling was opened, and there was another discharge of fetid material with gas, but no fæcal matter, nor evidence of perforation of the intestine. Recovery again took place promptly.

Dr. Keyes referred to another case, in which he opened what seemed to be a spontaneous abscess along the course of the descending colon, and which gave exit to a large quantity of gas and pus, but no fæcal matter, and there was no evidence of communication with the gut. The patient recovered promptly.

Dr. A. C. Post referred to a case in which he operated for abscess in the left groin, but differed from the ordinary abscess occurring in the right groin in that it had a very chronic course. The patient had been sick a number of months, and during that time the swelling increased and then diminished in size a number of times, and when he came under Dr. Post's observation there was deep-seated fluctuation. The layers of fascia were cautiously divided until the abscess was reached and exit given to purulent matter. From that time onward there had been a small discharge of fæcal matter. He had made efforts with the potential and the actual cautery, but had not succeeded in closing the fistula. It was the only case which he had seen in which there was a fæcal abscess in the left groin presenting the features of an abscess in the right groin.

Dr. T. M. Markoe recalled a case which he saw with the late Dr. Peugnet. A young lady, tolerably healthy, was attacked by dysenteric diarrhœa, after attending a picnic, and the attack was accompanied by pain in the right side of the abdomen that continued for a few days, and then gradually subsided, yet not ceasing entirely. After a few days a swelling appeared in the right flank that was exceedingly tender to the touch. A few days subsequently

Dr. Markoe saw the patient, it being about the tenth day after the beginning of the local symptoms, when he examined the abdomen in the region of the cæcum, and found it free from induration; but, above the anterior superior spinous process of the ilium, and extending upward to the free border of the ribs, there was an induration of considerable size, which was the seat of tenderness and obscure fluctuation. Dr. Markoe advised making an incision, which was done, but it was not carried sufficiently deep to enter the abscess, although the limits of safety were apparently reached. He heard nothing more of the case until two or three months afterward, when he saw the mother of the patient, who said that when the tent was removed from the wound on the following morning, a large discharge of fetid matter took place, and she convalesced rapidly.

Dr. Briddon referred to three cases in which resolution took place under the influence of purgative doses of calomel and castor-oil, leeching, and opium. With reference to operative cases, he had had four, and all had recovered. He also referred to one case in which resolution took place after the tumor had existed for a long time. The tumor was large, and the initial symptoms were those of an ordinary perityphlitic abscess. The patient was a boy, and he was treated by administering a cathartic, making local depletion, applying fomentations, and giving opium; but the mass remained hard, although the general symptoms subsided. Fluctuation was never detected, and the aspirator was used with negative results. The symptoms subsided entirely, and the boy regained his health. The tumor remained unchanged apparently for four or five months, when another febrile attack was developed with flexion of the limb and tenderness at the seat of the swelling. The tumor, however, was of about its original size, and there was no fluctuation. It was strictly isolated in the iliac fossa. The inflammatory excitement subsided again within a few days, and the boy recovered so as to play in the usual way. Three months afterward the tumor seemed unchanged with reference to size and consistence, and was an elevated convex mass occupying the entire iliac fossa. Dr. Briddon then began to suspect that the tumor was sarcomatous in character, but the termination proved that the diagnosis of abscess was correct. No opening was made; there was neither discharge of pus into the intestine or the bladder, but the mass gradually diminished in size, and at the end of a year had nearly disappeared.

Dr. Poore referred to a case in which the tumor slowly subsided and the patient recovered under the influence of fomentations and opium.

Dr. E. Mason remarked that he had had eighteen cases under observation. Of these thirteen were in adults and five among children. In three cases the abscess ruptured into the rectum; one of the patients was an adult and the other two were children. In five cases he opened the abscess, and death occurred in two; in one by rupture of the sac seventy-four days after the abscess was opened (see *Medical Record*, June 10, 1879); in the other the abscess was opened in the lumbar region, and the patient died of septicæmia. In two cases the patient had two attacks, and in one of them the abscess was opened twice at an interval of seventeen months. In four cases the patients were females. Three cases occurred in the same family, the father and daughter, and in the same family one other member died of the same disease.

Dr. Mason remarked that he had noticed a nodular mass in the groin for a long time in cases in which resolution had occurred—in one case, in particular, lasting for several months.

*Fracture of the external condyle.*—Dr. L. A. Stimson referred to the subject of fracture of the external epicondyle and epitrochlea that had already been discussed in the Society (see Minutes of Stated Meeting, March 9, 1880), and brought forward an important addition to the literature in cases reported by Dr. A. Coulon in "A Clinical and Practical Treatise on Fractures in Children," and published in 1861 (French).

*Plastic Operations on the Face.*—Dr. A. C. Post exhibited drawings which illustrated his method of performing two extensive plastic operations upon the face.

*Periosteal Sarcoma of the Tibia aud Fibula.*—Dr. C. K. Briddon presented a specimen accompanied by the following history:

Theodore Blumerloh, aged seventeen; single, of German extraction, by occupation office-boy. Family history: father died of phthisis; mother alive and in good health; other members in the enjoyment of perfect health, and no case of malignant disease known to have occurred in any of his ancestors.

Present illness: one year ago, whilst going down stairs, he twisted his left ankle. The accident was regarded as trivial, and did not prevent him following his usual occupation. Three months later a swelling gradually developed in, or in the immediate neighborhood of, the joint, for which he used some domestic remedies, and subsequently seeking professional advice, was directed to use an elastic bandage, which he did with benefit.

Three months ago, following exposure to cold and wet feet, the joint commenced to be painful. The pain subsided in a few days, but the swelling gradually increased, and on the 8th of April three incisions were made by his medical attendant. From one of the incisions made on the inside of the joint a fungous growth gradually presented.

On admission to Presbyterian Hospital (service of Dr. Briddon) the boy presented a very emaciated condition—tall in stature, pale and bloodless in appearance. His left ankle was involved in a swelling of huge proportions, elastic to the feel, and with large veins meandering over its surface. The inner side of this swelling was occupied by a large fungous mass 6 cms. in circumference. As stated above, the swelling was everywhere elastic, but nowhere could be detected any parchment crackling; glands below Poupart's ligament very slightly engorged, probably only hyperplasia of irritation. The diagnosis was osteo-sarcoma, and amputation through the knee-joint was advised and accepted by the patient. May 15, 1880, under the influence of ether, and with strict antiseptic precautions the amputation was made with anterior skin flap five inches, and posterior skin and muscle three inches in length.

The boy recovered well from the shock of the operation, but immediate union was marred by sloughing of the anterior flap to the extent of an inch and a quarter; but at the time of making the report he was doing as well as could be expected.

*Pathological Report.*—It was found, after a vertical section had been made through the leg, dividing the tibia, fibula, and astragalus, and cutting off the posterior portion of the os calcis, that there was a growth four inches in length encircling the lower end of the tibia and fibula, passing down over the internal and also external malleolus, as far as the line of articulation between the astragulus and os calcis. The growth itself did not properly implicate the ankle-joint. The lower articular surface of the tibia was normal, but the opposing surface of the astragalus, the cartilaginous covering was slightly eroded. Although the growth completely encircled both bones, there was slight evidence of the fan-shaped radiation that has been commonly observed in periosteal sarcomata. At the central portion of the tumor, for a space of one and a half inch the periosteal covering of the bone was wanting. At this point the growth was easily separated, leaving a rough eroded surface. The bones of the leg and foot were observed to be a little softer than normal, but otherwise exhibited no change The bone subjacent to the growth presented no material change. The cut surface of the growth was of a pinkish color, with mottlings of lighter and darker shades. It was very soft to the feel, and indeed almost gelatinous.

Microscopically, the growth throughout presented a very uniform appearance, being made up of large, rounded corpuscles of the connective-tissue series, embedded in a homogeneous matrix. Newly formed vessels were numerous. The tumor was, therefore, regarded as a sarcoma of the large, round celled variety and recurrence might be expected.—*Medical Record.*

